# Unraveling the therapeutic effects of mesenchymal stem cells in asthma

**DOI:** 10.1186/s13287-020-01921-2

**Published:** 2020-09-15

**Authors:** Fatemeh Mirershadi, Mahdi Ahmadi, Aysa Rezabakhsh, Hadi Rajabi, Reza Rahbarghazi, Rana Keyhanmanesh

**Affiliations:** 1 Department of Physiology, Faculty of Medicine, Tabriz University of Medical Sciences, Daneshgah St, Tabriz, 51666-14766 Iran; 2 Department of Physiology, Ardabil Branch, Islamic Azad University, Ardabil, Iran; 3 Drug Applied Research Center, Tabriz University of Medical Sciences, Tabriz, Iran; 4 Cardiovascular Research Center, Tabriz University of Medical Sciences, Tabriz, Iran; 5 Koc University Research Center for Translational Medicine (KUTTAM), Koc University School of Medicine, Istanbul, Turkey; 6 Department of Pulmonary Medicine, Koc University School of Medicine, Istanbul, Turkey; 7 Stem Cell Research Center, Tabriz University of Medical Sciences, Tabriz, Iran; 8 Department of Applied Cell Sciences, Faculty of Advanced Medical Sciences, Tabriz University of Medical Sciences, Daneshgah St, Tabriz, 51548-53431 Iran

**Keywords:** Stem cells, Asthma, Regeneration, Cellular and molecular mechanisms

## Abstract

Asthma is a chronic inflammatory disease associated with airway hyper-responsiveness, chronic inflammatory response, and excessive structural remodeling. The current therapeutic strategies in asthmatic patients are based on controlling the activity of type 2 T helper lymphocytes in the pulmonary tissue. However, most of the available therapies are symptomatic and expensive and with diverse side outcomes in which the interruption of these modalities contributes to the relapse of asthmatic symptoms. Up to date, different reports highlighted the advantages and beneficial outcomes regarding the transplantation of different stem cell sources, and relevant products from for the diseases’ alleviation and restoration of injured sites. However, efforts to better understand by which these cells elicit therapeutic effects are already underway. The precise understanding of these mechanisms will help us to translate stem cells into the clinical setting. In this review article, we described current knowledge and future perspectives related to the therapeutic application of stem cell-based therapy in animal models of asthma, with emphasis on the underlying therapeutic mechanisms.

## Background

It has been estimated that pulmonary diseases are the third leading cause of death worldwide [1]. The pathological changes that occurred in asthma are complex and accompanied by prominent airway conduit inflammation and obstruction, bronchiole epithelial metaplasia, and overproduction of mucus [2]. Ultimately, the existence of such conditions leads to airway hyperactivity and exaggerated reactions in response to numerous endogenous and endogenous stimuli [3]. Several reports have shown that different immune cell types, predominantly eosinophils, and Th were recruited to the asthmatic niche coincided with abnormal formation of ECM [4]. Along with these changes, terminal alveoli and air sacs also show excessive pathological remodeling which is indicated by the thickening of the interstitial wall [5]. Ultimately, the persistence of asthmatic changes contributes to epithelial cell detachment and shedding, per-bronchiolar cuffing, type 1 collagen synthesis, and progressive loss of oxygen and carbon dioxide interchange [6]. From a mechanistic standpoint, asthma is categorized into both allergic and non-allergic forms which correlate with dynamic production IgE antibodies. In allergic asthma, the immune system is hyperactive and responds adversely to diverse stimuli [7]. Pathological examinations have revealed that Th2 cells actively were recruited into bronchioles and released different cytokines such as IL-4, IL-5, and IL-13. The cytokines shift the activity of resident pulmonary cells, such as epithelial cells, fibroblasts, and smooth muscle cells, and other immune cells mainly mast cells, eosinophils, and IgE-producing B lymphocytes [8, 9]. Due to the inefficiency of current treatment protocols, many researchers and clinicians try to find safe and effective modalities in the alleviation and restoration of the pulmonary system after asthmatic disease. This review aims to scrutinize experiments related to the application of stem cells and underlying cellular and molecular mechanisms participating in the alleviation of asthmatic changes.

To date, several methods have been developed for the induction of bronchial asthma using different animal models. For example, cotton dust, OVA, *Ascaris lumbricoides* antigens, cockroach extracts (*Blatella germanica*), house dust mite extract (HDM) (*Dermatophagoides pteronyssinus*, *Dermatophagoides farina*), fungi, and molds (*Aspergillus fumigatus, Alternaria alternata*), ragweed, latex (*Hevea brasiliensis*), and bacterial lipopolysaccharide (LPS) are allergens commonly implicated in asthma development and exacerbations [10–13]. Besides, the application of Alum via intraperitoneal route followed by airway challenges induces an acute allergic response that will mask immune events that are only partially contributing to inflammation and airway hyper-responsiveness [12, 14]. Considering the previously published experiments, it becomes clear that OVA, extracted from a chicken egg, is a widely used allergen for the sensitization of bronchioles in different animal models solely because of all the immune tools are available and recruited to the pulmonary niche after OVA challenge [13]. OVA can be produced on large scales at a lower cost. However, each sensitization method has strengths and weaknesses. It has been shown that repeated airway exposures to OVA may contribute to immune tolerance and the induced airway inflammation is not quite like what happens in the human asthmatic lungs. Distinct properties of HDM such as appropriate immunogenicity, direct activation of innate cells, and intrinsic enzymatic activity, make it suitable to mimic asthma-like conditions [13]. Commensurate with these descriptions, the selection of certain allergen correlates with the number of replicates and using alone or in combination with other allergens [15].

## Application of stem cells in the asthmatic niche

Over the past decade, the use of stem cell-based therapies and bioengineering modalities has been extensively studied for the regeneration of lung diseases. There are a growing number of scientific reports conducted in terms of different stem cells application to promote either structural or functional pulmonary restoration with a focus on both differentiation capacity and paracrine activity [16]. In this regard, it seems that adult bone marrow stem cells, including different lineages such as hematopoietic stem cells (HSCs), mesenchymal stem cells (MSCs), and endothelial progenitor cells (EPCs) hold splendid promise for the healing of injured tissues. The bone marrow niche is an appropriate microenvironment for the dynamic growth of HSCs and EPCs, while MSCs could be isolated from different tissues. In keeping with this theme, the regenerative potential of multiple stem cell types has been previously assessed in different chronic pulmonary pathologies such as asthma, COPD, and broncho-pulmonary dysplasia [16] (Table 1). According to the released data, stem cells were administered via intravenous infusion, intraperitoneally, or transplanted directly into the pulmonary niche via intranasal and intra-tracheal routes [42, 43]. Delving a bit deeper, the best appropriate administration route and cell delivery methods have not been determined yet. Considering relative ease of doing the experiments, it is thought that cell delivery via the intra-tracheal pathway is logically preferred to the other routes. For instance, the possibility of cell bio-distribution and colonization to the non-pulmonary tissues will be decreased dramatically which in turn reduces the dose of transplant target cells. Also, the direct introduction of distinct cell types to the injury site could yield better regenerative outcomes [43]. Even specific anatomical regions in the pulmonary niche encompass specific stem cells that are generally inactive under the normal condition and further acquire regenerative potential during injuries to the epithelial layers. Several reports highlighted the existence of pluripotency and stemness features in bronchioalveolar stem cells, non-ciliated bronchiolar secretory cells (Clara cells), basal cells, alveolar type II pneumocytes, and submucosal gland stem cells [44, 45]. Inside the lungs, there are a fraction of stem cells, namely c-Kit^+^ cells, with highly regenerative potential and self-renewal capacity [46]. It is thought that a large amount of c-Kit^+^ cells from bone marrow and systemic circulation and notably massive recruitment of these cells into the pulmonary niche not only did not ameliorate the progression of pulmonary disease but also exacerbate the progression of pathological responses [20]. Overall, the results from different animal models of asthma confirmed the advantageous and potential benefits after transplantation of stem cells which coincided with a reduction of inflammatory reactions, immune cell recruitment, and regulation of Th1 to Th2 ratio, reduced collagen fiber deposition in the lung parenchyma, and changes in the dynamic synthesis of pro- and anti-inflammatory cytokines. Besides, structural changes and pathological remodeling of the blood-air barrier, epithelial metaplasia, and mucus-producing goblet cells are faded post-stem cell transplantation [47].

**Table 1:** List of some in vivo and in vitro studies on the effects of stem cells or cell products on experimental induced asthma

Stem cell type	Route of administration	Animal model and type of injury	Time of study	Outcome	Ref
Bone marrow-derived c-Kit+ cells	Intratracheally	Hyperoxia-induced lung injury in rats	15 days	Angiogenesis and pro-angiogenic factors ↑, aveolarization↑, apoptosis↓	[17]
Anti-c-Kit siRNA	Intravenously	OVA-induced allergic asthma in mouse	72 h after siRNA injection	Pulmonary infiltration of inflammatory cells (eosinophils and lymphocytes)↓, IL-4 and IL-5↓	[18]
Anti-c-Kit siRNA	Intranasal	OVA-induced allergic asthma in mouse	72 h after siRNA injection	SCF, IL-4, and IL-5↓, eosinophil infiltration↓	[19]
c-Kit+ cells	Intratracheally	OVA-induced allergic asthma in mouse	10 days	Inflammation ↓, airway remodeling, and function↑	[20]
Bone marrow MSCs	Intravenously, Intratracheally	OVA-induced allergic asthma in mouse	More than 10 days	Th2 and Th17 cytokines↓, IgE↓, eosinophilia↓	[21]
Bone marrow MSCs and CM	Intratracheally	OVA-induced allergic asthma in rats	14 days	MSC-treated rats: neutrophili and neutrophilia↓, CD3^+^/CD4^+^↓, IL-10↑, IL-4↓	[22]
Bone marrow MSCs and CM	Intravenously	OVA-induced allergic asthma in rats	14 days	CD3^+^/CD4^+^↑, CD3^+^/CD8^+^↓, immune cells infiltration↓ (the therapeutic effects were more higher than CM)	[23]
Adipose-derived MSCs	Intratracheally	OVA-induced allergic asthma in mouse	ND	Airway responsiveness↓, lymphocytes infiltration↓, lgE, IL-1β ↓, IL-4 ↓, IL-17F↓, IL-10↑, RORγ↑, CD4^+^CD25^+^Foxp3 Treg cells↑	[11]
Adipose-derived MSC	Intravenously	Feline chronic allergic asthma	More than 4 months	Airway eosinophilia and responsiveness→, bronchial wall thickening ↓	[24]
Bone marrow mononuclear cells	Intratracheally	OVA-induced allergic asthma in mouse	7 days	Alveolar collapse↓; bronchoconstriction↓; fibrosis↓; IL-4, -5, and -13↓; IFN-γ↑; TGF-β↑	[25]
iPSC-derived MSCs	Intratracheally	OVA-induced allergic asthma in mouse	4 days	Connexin 43-mediated mitochondrial transfer↑, epithelial cells death↓	[26]
Human umbilical cord blood-derived MSCs	Intravenously	OVA-induced allergic asthma in mouse	29–31 days	IL-4, IL-5, and IL-13↓; IgE and IgG1↓; bronchial hyper-responsiveness↓; eosinophil infiltration↓	[27]
iPSCs, and mesenchymoangioblast-derived MSCs	Intranasal and Intravenously	OVA-induced allergic asthma in mouse	14 days	TGF-β1↓; airway/lung fibrosis↓; collagen-degrading gelatinase ↑	[28]
Human ESC-MSCs	Intravenously	OVA-induced allergic asthma in mouse	20 days	Th2 cells and eosinophils↓; Treg↑; periodic acid–Schiff positive cells↓; CD4^+^CD25^+^FOXP3^+^ cells↑; IL-4, IL-5, and IL-13↓; *Ccl11, Ccl24, Il13, Il33,* and *Ear11* expression ↓	[29]
Bone marrow, umbilical cord, and adipose-derived MSCs	Intravenously	OVA-induced allergic asthma in mouse	7–10 days	Eosinophil↓; IL-4, IL-5, and IL-13↓; INF-γ↑; IL-10 producing macrophages↑	[30]
MSC-derived exosomes	In vitro	Target cells: asthmatic peripheral mononuclear cells	24 h	IL-10 and TGF-β↑, proliferation of CD4^+^CD25^+^FOXP3^+^ cells↑	[31]
MSCs CM	In vitro	GM-CSF-induced asthmatic changes in 3 T3 murine airway fibroblast cells	14 days	Collagen types I, III↓; hyaluronan↓	[32]
MSCs	Retro-orbital	OVA-induced allergic asthma in mouse	4 weeks	Hyaluronan↓, airway inflammation↓	[32]
Adipose-derived MSCs	Intravenously	OVA-induced allergic asthma in mouse	12 days	IDO, TGF-β, and PGE2↑ (IL-4, IL-5, and IL-13↓); IFN-γ↑; IL-10↑	[33]
Human placenta MSCs	Intravenously	OVA-induced allergic asthma in rats	22 days	Notch3 and delta-4↑; notch-1, -2 and jagged-1↓; IgE, Th2 cytokines↓	[34]
iPSC-derived MSCs	Intravenously	OVA-induced allergic asthma in mouse	55 days	Fibrosis and α-SMA↓, TGF-β1↓, phosphorylated Smad2/3 expression↓	[35]
Adipose tissue MSC-derived extracellular vesicles	Intravenously	OVA-induced allergic asthma in mouse	7 days	TGF-β↓, fibrosis↓, inflammation↓, bronchiolar Siglec-F^+^ eosinophils↓, eotaxin↓, CD3^+^ CD4^+^ cells↓, CD4^+^CD25^+^Foxp3^+^ cells↑	[10]
Bone marrow MSCs	Intravenously	OVA-induced allergic asthma in mouse	7 days	Pulmonary oxidative stress↓, and nitrotyrosine↓	[36]
Adipose-derived MSCs and bone marrow-derived MSCs	Intratracheally	HDM-induced allergic asthma in mouse	3–7 days	Bone marrow MSCs: IL-10↑, the influx of eosinophils and B cells ↓, alveolar macrophage inflammatory response↓, lung function, and remodeling→, adipose-derived MSCs were ineffective	[15]
Adipose-derived MSCs	Intravenously	HDM-induced allergic asthma in mouse	3 days	Inflammation↓, Th1 cytokines↓, hyper-responsiveness →, contractile tissue→, cell integration, and differentiation →	[37]
Bone marrow-derived MSCs	Intravenously	HDM-induced allergic asthma in mouse	8–10 days	Airway responsiveness↓, bronchial contraction ↓, inhibitory type 2 muscarinic receptor↑, phagocytosis of MSCs by local macrophages, macrophage M2 suppressive phenotype↑	[38]
Human iPSC-MSCs	Intravenously	Neutrophilic airway inflammation induced by LPS and OVA in mouse	4–48 h	Th cells (Th17)↓, Th cells-associated cytokines↓, neutrophilic airway inflammation↓, p-STAT3↓, GATA3↓, RORγt↓, iPSC-MSCs differentiation into Th cells↑	[39]
Adipose-derived MSCs	Intravenously	HDM-induced allergic asthma in mouse	7 days	IL-3 and IL-4↓, BALF CD4^+^ T cells, and Eosinophils↓, Fibrosis↓, TGF-β↓, α-actin↓	[40]
Bone marrow-derived MSCs	Intravenously	*Aspergillus fumigatus* hyphal extract-induced asthma in mouse	76–78 days	Th17-mediated airway inflammation↓, T regulatory cells →, airway hyper-responsiveness↓, BALF Th2, and Th17 soluble mediators↓	[41]

*ND*, non-determined; “↑”, increase; “↓”, decrease; “→”, ineffective

## Application of MSCs in asthma

In a review of previously published experiments, MSCs have been extensively applied in the alleviation of asthma in different animal models more than other types of stem cells [48]. Many researchers showed that MSCs could proliferate for multiple passages which allow for large-scale production of these cells for different regenerative medicine applications in animal models of asthma. Based on a scientific document, it has been shown that MSCs are capable of suppressing inflammatory response and pathological remodeling in the asthmatic context [47, 49]. Based on conducted experiments, MSCs were transplanted to the asthmatic animals at the range from 1 × 10^6^ to 5 × 10^7^ [50, 51]. According to histological examination, these cells easily migrate toward inflammatory sites in response to cytokine concentration gradients after systemic or local administration. It can be claimed that the production of different factors and cytokines triggers MSCs activation. In vitro pre-treatment of bone marrow-derived mesenchymal stem cells with sera from asthmatic mice increase immunomodulatory properties in allergic asthma [52]. It seems that the positive therapeutic effects of MSCs are mainly done by releasing an array of factors in a paracrine manner which modulates the cell-based and humoral immune responses compared to differentiation potential and juxtacrine activity [43]. In support of this statement, several papers were published that the majority of transplant MSCs are cleared from the pulmonary niche after few days possibly through phagocytosis by alveolar macrophages or apoptosis pathways, raising the question of how they prompt such long-lasting immunosuppressive effects [53]. The activity of recipient immune cells, cytotoxic T cells, promotes MSCs apoptosis via perforin-dependent mechanism [54]. Although it may seem that the decrease of transplanted MSCs by immune rejection could diminish regenerative outcome this phenomenon is done in antibody- and MHC-free manner [54]. Surprisingly, the possible apoptotic death of transplanted MSCs in the asthmatic niche could in part, but not completely, regulated local cellular and humoral immunity via the regulation of phagocytes recruited to the pulmonary tissue [55]. Besides, an elevated ROS generation and enhanced pro-inflammatory cytokines could accelerate functional MSCs depletion at the site of inflammation by eliminating trans-differentiation capacity, self-renewal, and prompt aging [56]. Despite these limitations after the introduction of MSCs to the asthmatic niche, MSCs potentially possess magnificent immunomodulatory capacity without provoking immunogenic responses. MSC secretome harbors diverse factors and cytokine could regulate the functional activity of T and B lymphocytes, dendritic cells, and natural killer cells [57]. Even in the presence of TNF-α and IFN-γ, MSCs can acquire immunosuppression phenotype and immunomodulatory properties. It seems that the production of indoleamine 2,3-dioxygenase and prostaglandin E_2_ is actively involved in this phenomenon [58].

Several experiments revealed that MSCs exhibited a different restorative capacity based on isolation from various tissues. Also, the MSC subtype possesses a different multipotential capacity [59]. Bone marrow is a primary and well-known source for the isolation of MSCs. However, alternative sources for MSCs isolation in order of importance and number of conducted studies are adipose tissue, blood, umbilical cord blood, skeletal muscle, tendons, lung, etc. [60]. In addition to differences in the therapeutic capacity of MSC subtype, experiments have shown distinct immunomodulatory properties for multiple MSC types. For instance, it has been elucidated that MSCs isolated from adipose and placenta tissues elicited robust immunomodulation in experimental allergic asthma compared to bone marrow MSCs [61, 62]. Lung and umbilical cord blood MSCs have short-lasting persistence in inflamed sites compared to bone marrow MSCs administrated intravenously [63, 64]. Li et al. claimed that the introduction of placental-derived MSCs in OVA-sensitized rats, upregulated IL-10, reduced IL-17, and blunted Th17 to Treg ratio [65]. Recent data examining the anti-asthmatic properties of placental MSCs within the pulmonary niche showed the reduction of eosinophils in bronchoalveolar lavage fluid and suppression of IgE and IL-4. Along with these changes, the proliferation of goblet cells, and synthesis of mucus closed to near-normal levels and lymphocyte polarization toward Th2 was interrupted [34].

It is noteworthy that various MSC subtypes could exhibit diverse regenerative potential in the asthmatic pulmonary niche. As abovementioned, it seems that the source of MSCs could alter persistence in the inflammatory asthmatic niche correlated with the ability to express adhesion molecules, distinct integrins. MSC subtype isolated from the multiple tissues possess different global gene expression profiles and paracrine activity which may contribute to diverse regenerative outcomes [66]. In support of this statement, it has been shown that intra-tracheal administration of mouse MSCs from three different tissues such as adipose tissue, bone marrow, and lungs modulated inflammatory response and structural remodeling with different outcomes in OVA-induced asthma model, possibly via distinct secretome profile [66]. Abreu and colleagues highlighted a superior paracrine activity of bone marrow MSCs in comparison with adipose-derived counterparts [66]. Hence, it seems logical to select an appropriate MSC type with a special multipotentiality regarding the entity of pulmonary injury.

## Mechanisms involved in the therapeutic effects of MSCs in experimental asthma models

### Immunomodulation

**Fig. 1:**
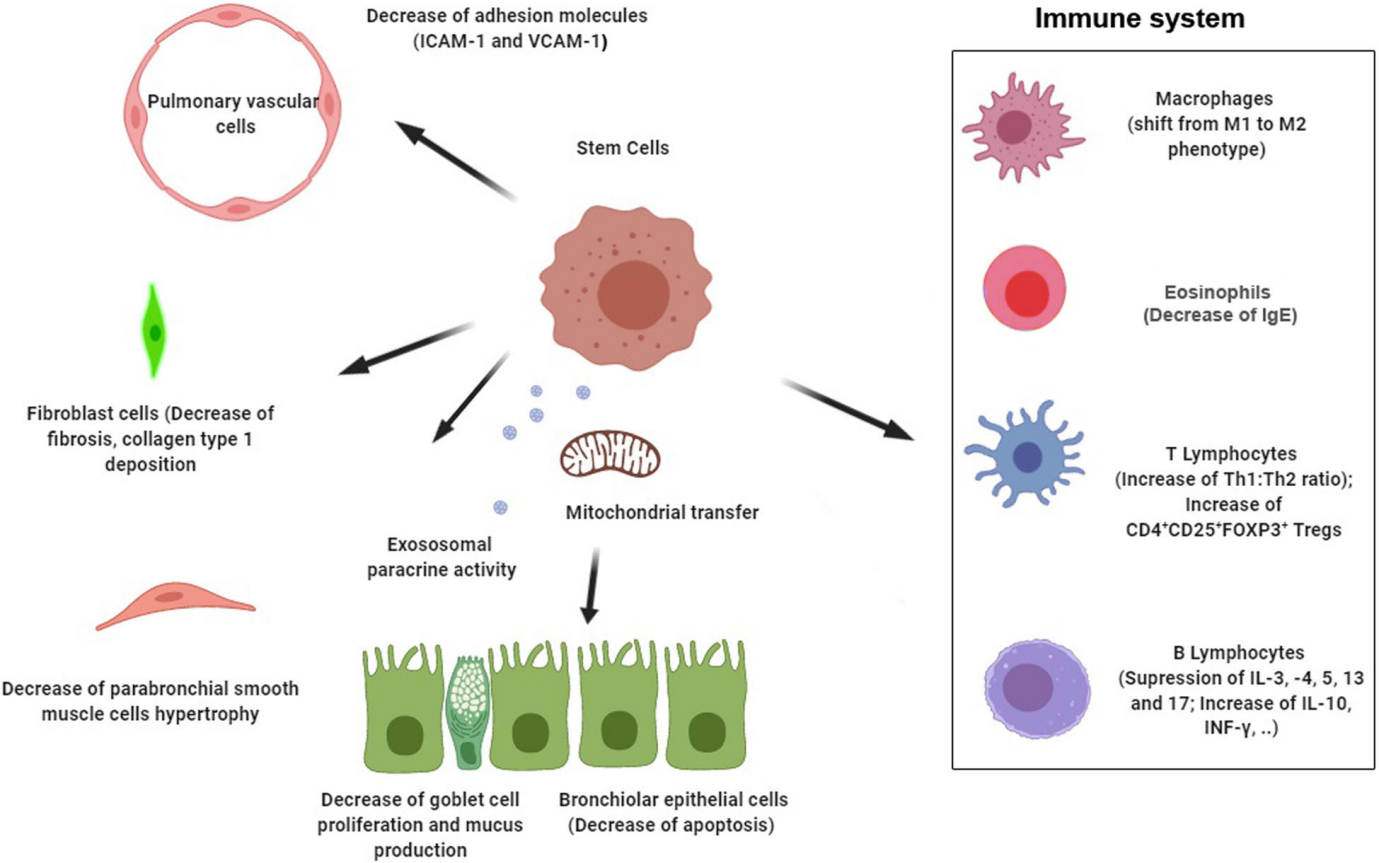
Pleiotropic effects of stem cells on pulmonary asthmatic niche via different mechanisms

As abovementioned, MSCs have been extensively applied in several experimental studies in asthma. It seems that the therapeutic properties of MSCs are mainly correlated with the immune system regulation at the site of inflammation [43, 63, 64, 67]. MSCs are eligible to regulate Th2 to Th1 ratio, synthesis of interleukins such as 4, 5, and 13; IgE; and mucus after introduction into the asthmatic niche. By increasing TGF-β and IFN-γ, MSCs can abort untamed allergic responses [67–69]. These events coincide with the increase of CD4^+^CD25^+^ FoxP3^+^ Treg cells, IL-10 in bronchioalveolar discharge, a decrease of mast and goblet cells, suppression of nitrosative stress, and inhibition of phagocytic activity in alveolar macrophages and [38, 67, 70] (Fig. 1). There is a close reverse relationship between the eosinophil population in bronchoalveolar lavage and Treg cells which seem critical for the protective impact of MSCs [71]. Most experiments implied that the control of Th2 lymphocytes and relevant allergic reactions could be an efficient strategy for the control of asthmatic injuries [67–69]. Notably, different types of MSCs transplantation have different time-dependent therapeutic outcomes. Due to non-specific bio-distribution and the problem associated with the capacity to cross the blood-air sac barrier, intravenous administration possibly leads to inadequate MSCs recruitment to the asthmatic niche. Despite these limitations, this approach is recommended in unstable conditions [72]. Some authorities claimed that approximately 4- to 5-fold doses of MSCs is required to yield the same therapeutic outcome applicable to results by local administration [72]. Based on previously published data, direct intra-tracheal delivery of whole bone marrow mononuclear cells yielded more cells trapped inside the lung parenchyma in comparison with the systemic route. Both modalities resulted in a similar immunomodulatory capacity of MSCs [25]. It has been shown that intraperitoneal administration of bone marrow MSCs is the potential to modulate the allergic asthma reaction. Soon after injection into the peritoneal cavity, these cells easily migrate to the pulmonary niche and exert immunomodulatory capacity [73, 74].

If we assume that the therapeutic capacity of transplant MSCs is mainly done via paracrine activity, thus, it is logical to transplant distinct cell types to the asthmatic niche locally rather than via a systemic route [28]. Of course, we must be aware that the local administration is per se invasive, expensive due to surgical procedure and postoperative care. Due to mechanical pressure and inflammatory tissue conditions, a fraction of transplant MSC dies soon after injection into inflamed sites. Despite these limitations, fewer MSCs are needed in local delivery to accomplish therapeutic efficacy. Calling attention, paracrine activity is just efficient in a short distance after close interaction of pulmonary resident cells with MSCs in proximity [75]. Considering these limitations, the repeated dose of MSCs could, if not completely, circumvent disadvantage related to systemic administration in the asthmatic niche [76]. Even though, a repeated dose of MSCs increases the risk of ectopic non-specific overgrowth in different tissues except the lungs [77]. It should be in mind that most studies in animal models reported the efficiency of MSCs in the alleviation of asthmatic changes soon after initial sensitization. Some researchers used episodic allergen exposure to the stabilized asthma-like condition [24]. However, there are few long-term follow-up studies related to the therapeutic effects of MSCs asthmatic animals. Commensurate with these descriptions, there was no basis for the statement that MSCs could completely restore or retrieve the asthmatic lung function. For example, Trizl and co-workers performed six intravenous administrations of MSCs bi-monthly in asthmatic cats and followed up for 1 year [24]. They declared that MSCs failed to suppress inflammation of airway conduits in terms of eosinophil number and bronchiolar hypersensitivity. In another study done by the same research group, five systemic administrations of MSCs showed an anti-inflammatory response at day 130 while data represented the lack of prominent inflammation suppression by month 9 [78]. The scientific rationale for these data could correlate with the fact that bona fide MSCs exert therapeutic effects only short periods after transplantation via differentiation, juxtacrine, and paracrine mechanisms before their death.

### Multiple mechanisms action of MSCs in asthmatic niche

In addition to the immunomodulatory capacity of MSCs, growing evidence has proposed the existence of multiple mechanisms, such as trans-differentiation capacity, cell fusion, mitochondrial transfer, and paracrine activity done by microvesicles and exosomes, in these cells to alleviate asthma-related pathology [79] (Fig. 1). As aforementioned, multiple experiments confirmed the existence of factors and cytokines in MSCs secretome which are packed inside extracellular nano-sized vesicles, namely exosomes and transported to the target cells. Additionally, the direct differentiation potency of MSCs has been neglected previously by reports which are possibly due to the enhanced MSCs death at the site of transplantation [22, 80]. A study conducted by Spees and co-workers showed that the simultaneous culture of human MSCs with heat-injured pulmonary epithelial cells promoted differentiation toward epithelial-like lineage [81]. Based on the facts from experiments, it would not be an exaggeration to mention that paracrine activity is the main suggested therapeutic bioactivity of MSCs in the asthmatic niche [82]. These cells have inherent capacity to release 40–200 nm nanoscale exosomes which harbor multiple anti-inflammatory factors that could regulate the function of immune cells [83]. Interestingly, these nanoparticles easily spread in the bio-fluids, are stable, and survive in a harsh milieu when even the source cells could not survive [84]. On this basis, Cruz and co-workers claimed that systemic injection of conditioned media (CM) or extracellular vesicles harvested from mouse and human MSCs are equally effective in the regulation of Th2/Th17-associated asthma hypersensitivity and inflammation in a mouse model of mycosis [85]. Concerning the fact that some regenerative effects of MSCs are done via releasing soluble effectors, thus, MSC-free therapy such as CM and exosomes could be an alternative due to easy storing and handling. By using these approaches, it is less likely to see cellular emboli, tumorigenesis, and unwanted immune responses after transplantation [86]. According to data from a study conducted by Keyhanmanesh et al., they reported that three times the systemic injection of MSC CM inhibited hallmark of asthma indicated by suppression of IL-4 and increase of INF-γ. The results were accompanied by the upregulation of T-bet and downregulation of GATA-3 in the asthmatic rats [76], due to a large number of factors inside exosomes exhibiting pleiotropic effects. For instance, Rahbarghazi et al. demonstrated that the intra-tracheal administration of either rat bone marrow MSCs or CM could suppress the Th2-associated activity via suppression of IL-5 and activation of IL-12. They also found that the expression of adhesion molecules such as intercellular adhesion molecule-1 and vascular cell adhesion molecule-1 significantly decreased which per se decreased immune cell recruitment to the pulmonary tissues [48]. In contrast, Ahmadi and colleagues reported the inefficiency of rat MSC CM on the modulation of inflammation in OVA-induced asthma [23]. The reason for contradictory results could correlate with the time and route of administration, and total dose [66, 87]. Additionally, short activity and rapid distribution of factors after transplantation compared to cell injection could be logic for transient therapeutic effects of CM and exosomes. Aside from the fact that CM and exosomes are integral to the paracrine activity of MSCs in injured sites, more investigations are highly demanded to address underlying mechanisms.

Some data showed that MSCs promote tissue regeneration via mitochondrial transfer is a response to external stimuli. The critical role of gap junctional channel, tunnel tube formation and Rho-GTPases such as Miro1 were previously confirmed by which mitochondrial mass was transferred to the damaged cells [88, 89]. In this regard, it has been shown that connexin-43 GJC^+^ MSCs retrieved epithelial cell bioactivity after mitochondrial transfer in lipopolysaccharide-associated acute pulmonary inflammation. Islam and co-workers found that the suppression of connexin-43 was interrupted by mitochondrial transfer from MSCs to epithelial cells [90]. It seems that the phenomenon of mitochondrial transfer is effective in the alleviation of other pulmonary injuries. For example, Li et al. confirmed the therapeutic effect of bone marrow MSCs against rat COPD induced by cigarette smoke [91]. In a recent study, it has been shown that the intra-tracheal administration of induced pluripotent stem cell-derived MSCs improved mitochondrial dysfunction in epithelial cells via mitochondrial transfer via connexin-43 [26]. Mitochondrial transfer is efficient to slow down the procedure of apoptotic changes in epithelial cells [92]. In addition to the mitochondrial donation, multiple factors released by MSCs could inhibit the apoptosis signaling pathway either in a non-mitochondrial or mitochondrial-dependent manner [93]. Besides, other cell-protective mechanisms, namely autophagy, actively could alter the development of asthmatic remodeling. For instance, it has been shown that intravenous administration of MSCs reduced inflammation in the pulmonary microvascular system via engaging autophagy-related effectors during ischemia/reperfusion in the model of mouse by inhibiting miR-142a-5p in endothelial cells [94, 95].

Unraveling the regenerative effects of MSCs and other stem cell types could be done in both protein and gene levels. The discovery of miRNAs and other factors function is a de novo strategy for the reduction of asthmatic changes [96]. As implied by previous experiments, molecular examination confirmed the alteration of miRNAs during asthmatic changes. Therefore, elucidation of miRNAs and relative changes could be a reliable tool for monitoring the progression of asthma [97, 98]. Further investigations in animal asthma models showed the potency of MSCs and induced pluripotent stem cell-derived MSCs to modulate the expression of pro-inflammatory miRNAs, such as miRNA-155, -133, mmu-miR-21a-3p, and mmu-miR-449c-5p coincided with the induction of miRNA21 and mmu-miR-496a-3p [97, 99, 100]. For example, previous studies showed that MSCs could alter the phenotype and bioactivity of different cells via horizontal transfer of genetic data such as miRNAs and mRNAs [101]. It has been shown that exosomes are the major players in the paracrine activity and transfer of genetic materials and factors from MSCs to the immune cells [101]. The exposure of M1 pro-inflammatory macrophages to the MSC-derived exosomes induced polarization toward M2 anti-inflammatory macrophages [102]. As previously mentioned, the overproduction of Th2-derived cytokines, such as IL-4, IL-5, IL-9, and IL-13, is associated with dysregulated immunity in asthma [103]. It has been shown that the application of MSCs in different asthmatic models could decrease inflammatory response by altering the levels of Th2-derived cytokines and miRNAs associated with the function of these cells [21]. The interaction of microbial pathogen-associated molecular patterns (PAMPs) with pulmonary epithelial cells and immune cells via toll-like ligand receptors in asthmatic niche leads to the production of cytokines and chemokines [104]. Toll-like ligand receptors were also expressed on the surface of different stem cells such as MSCs and endothelial progenitor cells [105, 106]. It seems that the simultaneous stimulation of MSCs and immune cells suggests the putative role of MSCs in controlling the activity of immune cells. The activation of toll-like ligand receptors by PAMPs could frustrate pulmonary macrophages and release a large content of chemokines such as CXCL8 and CXCL11 which in turn increase the migration of MSCs toward the site of asthmatic niche. Also, the presence of MSCs suppresses the activity of microbes by producing antibacterial proteins [107]. It has shown that MSCs can suppress the function of complement cascade by releasing complement inhibitors such as factor H, leading to the inhibition of C3 and C5 convertase [108]. According to different experiments, several miRNAs have critical roles in the inflammation of airway conduit, including miRNA-126, miRNA-let-7, and miRNA-155 [97]. In a study performed by Kuo and co-workers, the therapeutic potential of MSCs has been proved to reduce stretch-induced inflammatory miR-155 in pulmonary bronchiolar epithelial cells by downregulating miR-155 [109]. Data showed that MSCs are promising cell sources to alter the expression of miRNAs in immune cells and pulmonary tissue to reduce the inflammatory condition.

Despite numerous advantages of MSCs application, there are very few reports regarding MSC side effects in pulmonary disease. For instance, it was shown that allogenic transplantation of MSCs in patients with idiopathic pulmonary fibrosis did not cause serious clinically and laboratory abnormalities [110]. However, the long-term follow-up of these patients revealed a total number of 2 deaths per 9 MSC-treated cases because of disease exacerbation [110]. Clinical trials conducted already by local investigators in different countries showed that transplantation of MSCs was appropriately tolerated and only a limited number of side effects were observed due to uncontrolled suppression of immune cells. Besides trans-differentiation of transplanted to undesired cell types, the progression of tumor-like cells and possible metastasis to remote sites are the main challenging issues [110]. Attention should be made to interpret the immunomodulatory properties of MSCs after transplantation under acute and chronic inflammation. It has been shown that the administration of allogenic MSCs increased alveolar macrophage activity a few hours after transplantation via the intravenous route indicated by enhanced MCP-1, CXCL-1, and IL-6 production [111]. To increase the survival rate and modulatory effect of transplanted MSCs, the simultaneous administration of mycophenolate mofetil was performed from the time of cell administration onwards. This strategy inhibits the accumulation of reactive T cells and allogeneic rejection [112]. There is still a long way to go to confirm the therapeutic outcomes of MSCs in different pulmonary allergic diseases.

## Xenogeneic transplantation of human MSCs into an animal model of asthma

Despite the existence of inherent species variation in MSC function, some experiments conducted xenogeneic lung transplantation models in animals using human MSCs [113–115]. Similar to animal MSCs, human MSCs exhibited potent immunomodulatory properties either in the acute or chronic asthma mouse model [114, 115]. It has been elucidated that typical hallmarks of asthma were mostly subsided after transplantation of human MSCs, isolated from bone marrow, adipose tissue, and umbilical cord, in the mouse model [30]. Systemic injection of human bone marrow MSCs via tail vein induced pulmonary macrophage polarization toward M2 type via the promotion of the TGF-β/Smad signaling pathway [116]. Interestingly, xenogeneic transplantation of human MSC retro-orbital administration in mice diminished the hyaluronan mucus in the chronic asthma model [32]. These findings support the assumption that autologous, allogeneic, and xenogeneic transplantation of MSCs could promote anti-inflammatory outcomes via engaging different mechanisms. Regarding massive genetic heterogeneity in allogeneic and xenogeneic models, these cells are, although not fully, able to exert regenerative outcomes.

## Clinical trials

According to the promising results of animal studies, some efforts have been made to investigate the paracrine and juxtacrine effect of MSCs on the human counterpart of asthma. By March 2020, clinical trial results (conducted in URL: https://clinicaltrials.gov) represented about 9 clinical trials to deal with asthma in the patients (Table 2). Of the nine clinical trials, two ongoing examinations evaluated the efficacy of MSCs in asthmatic patients. In a clinical trial conducted by the University Of Miami Miller School Of Medicine, the therapeutic effects of allogeneic MSCs were examined intravenously and patients were followed for 12 weeks. In the second study performed by Punta Pacifica Hospital of Panama City, intranasal administration of human umbilical cord MSC-derived trophic factors was evaluated in adult asthmatic patients.

**Table 2:** The list of clinical trials concerning asthma documented up to March 2020

Status	Study title	Conditions	Interventions	Phase	Number enrolled
Active, not recruiting	Allogeneic human cells (hMSCs) via intravenous delivery in patients With Mild asthma	Asthma	Biological: hMSCs	1	6
Active, not recruiting	Safety and feasibility study of an intranasal mesenchymal trophic factor for the treatment of asthma	Asthma	Biological: trophic factors from umbilical cord mesenchymal stem cells	1 and 2	20
Completed	Study to evaluate the effect of benralizumab on allergen-induced inflammation in mild, atopic asthmatics	Asthma	Biological: benralizumab, other: placebo	Phase 3	46
Completed	A pilot study for cell-based therapies in patients with asthma	Asthma, allergic rhinitis	Other: blood donation	Not applicable	20

## Conclusions and future perspectives

Overall, MSC delivery could diminish inflammation of lungs and airway conduits in the asthmatic animal models. The therapeutic effects of MSCs are done by involving different molecular and cellular pathways related to immunomodulation, mitochondrial donation, and protection against different pathways leading to cell death such as apoptosis and oxidative stress. It is highly recommended to establish diverse basic experiments and clinical trials to address the precise underlying mechanisms of MSCs therapy in asthmatic subjects. Long-term monitoring of asthmatic patients who received MSCs could carefully highlight the possible ineffectiveness and side effects before making a solid decision about cell-based therapies.

Defining the exact mechanism of MSC-therapy in the asthmatic condition is mandatory before the advent of cell therapy as an alternative modality in the clinical setting. The long-term outcomes and survival of locally or systemically administrated MSCs should be determined. The possible side effects of in vitro expansion of MSCs should be determined. We suggest future investigations need to find appropriate dosing and administration routes. Meanwhile, the exact bioactivity of MSCs is still unclear under acute and chronic inflammation.

## Data Availability

All data generated or analyzed during this study are included in this published article.

## Abbreviations

COPD: Chronic obstructive pulmonary disease
CM: Conditioned media
ESCs: Embryonic stem cells
ECM: Extracellular matrix
GJC: Gap junction
HSCs: Hematopoietic stem cells
HDM: House dust mite extract
iPSCs: Induced pluripotent stem cells
IFN-γ: Interferon-gamma
IL: Interleukin
LPS: Lipopolysaccharide
MSCs: Mesenchymal stem cells
EPCs: Endothelial progenitor cells
miRNAs: MicroRNAs
OVA: Ovalbumin
siRNA: Small interference RNA
SCF: Stem cell factor
Th: T helper lymphocytes
TGF-β: Transforming growth factor-beta
TNF-α: Tumor necrosis factor-alpha
